# mCherry fusions enable the subcellular localization of periplasmic and cytoplasmic proteins in *Xanthomonas* sp.

**DOI:** 10.1371/journal.pone.0236185

**Published:** 2020-07-30

**Authors:** Michelle Mendonça Pena, Doron Teper, Henrique Ferreira, Nian Wang, Kenny Umino Sato, Maria Inês Tiraboschi Ferro, Jesus Aparecido Ferro

**Affiliations:** 1 Agricultural and Livestock Microbiology Graduation Program, School of Agricultural and Veterinarian Sciences, São Paulo State University (UNESP), Jaboticabal, SP, Brazil; 2 Department of Microbiology and Cell Science, Citrus Research and Education Center, University of Florida, Lake Alfred, FL, United States of America; 3 Department of Biochemistry and Microbiology, Biosciences Institute, São Paulo State University (UNESP), Rio Claro, SP, Brazil; 4 Department of Technology, School of Agricultural and Veterinarian Sciences, São Paulo State University (UNESP), Jaboticabal, SP, Brazil; USDA/ARS, UNITED STATES

## Abstract

Fluorescent markers are a powerful tool and have been widely applied in biology for different purposes. The genome sequence of *Xanthomonas citri* subsp. *citri* (*X*. *citri*) revealed that approximately 30% of the genes encoded hypothetical proteins, some of which could play an important role in the success of plant-pathogen interaction and disease triggering. Therefore, revealing their functions is an important strategy to understand the bacterium pathways and mechanisms involved in plant-host interaction. The elucidation of protein function is not a trivial task, but the identification of the subcellular localization of a protein is key to understanding its function. We have constructed an integrative vector, pMAJIIc, under the control of the arabinose promoter, which allows the inducible expression of red fluorescent protein (mCherry) fusions *in X*. *citri*, suitable for subcellular localization of target proteins. Fluorescence microscopy was used to track the localization of VrpA protein, which was visualized surrounding the bacterial outer membrane, and the GyrB protein, which showed a diffused cytoplasmic localization, sometimes with dots accumulated near the cellular poles. The integration of the vector into the *amy* locus of *X*. *citri* did not affect bacterial virulence. The vector could be stably maintained in *X*. *citri*, and the disruption of the α-amylase gene provided an ease screening method for the selection of the transformant colonies. The results demonstrate that the mCherry-containing vector here described is a powerful tool for bacterial protein localization in cytoplasmic and periplasmic environments.

## Introduction

*Xanthomonas citri* subsp. *citri* (*X*. *citri*) is a Gram-negative, rod-shaped pathogenic bacterium that causes Asiatic citrus canker (ACC) in Rutaceous plants [1]. ACC is one of the most serious problems for the citrus industry and since it is present in both Northern and Southern Hemispheres, many efforts have been dedicated to eradicating the disease [2]. Although there is no effective treatment, the eradication of symptomatic trees combined with recurrent sprays of copper-based bactericides is one of the control methods [3, 4], but still insufficient to keep the citriculture free of the disease.

One of the most powerful strategies to defeat a phytopathogen disease is to understand the mechanism used by the pathogen to break down the host defense barriers. *X*. *citri* genome was published many years ago [5], and since then a strong effort has been done to understand the molecular pathways used by this bacterium during the infection process [6–10]. The virulence of *X*. *citri* is dependent on the translocation of effector proteins into the host cell through the type III secretion system, allowing the bacteria to cause disease [11]. The role of these virulence proteins and their cellular localization can provide powerful information to understand the bacteria biology and to develop potential antimicrobial compounds.

Although there are many ways to characterize the function of a gene, new tools need to be developed to improve and make the characterization more accurate. Protein function could be investigated in part by fusing the protein to fluorescent proteins with subsequent determination of the subcellular localization of the fusion protein [12]. Fluorescent proteins have the advantage of not requiring a substrate for fluorescence, which usually make them free of solubility, toxicity, or permeability problems [13]; however, sensitivity to photobleaching is still a big challenge [12]. One of the first works using fluorescent protein as a cytological reporter was done using green fluorescent protein (GFP) [14]. After this, problems with the export of functional GFP to the periplasmic region were reported [15]. Alternatively, other fluorescent proteins, like mCherry, were developed, which displayed correct folding and function in the periplasmic space [15, 16]. MCherry is a monomeric red fluorescent protein with an excitation/emission wavelength at 587/610nm, respectively. This protein is a homolog derived from DsRed, originally isolated from *Discosoma* sp. (cnidarian) and has been widely applied as a fluorescent protein label in prokaryotes and eukaryotes [17–19].

Here we report on the construction of an integrative vector, pMAJIIc, with a distinguishable fluorescent marker–mCherry–that can be used to localize proteins in the periplasm of *Xanthomonas* sp. Fluorescence emission could be easily differentiated in *X*. *citri* cells and the bacterial micrography allowed the visualization of its cytoplasmic and periplasmic proteins. The pMAJIIc vector is stable and does not affect bacterial development, virulence, or pathogenicity.

## Material and methods

### Bacterial strains, plasmids, and culture conditions

The strains and plasmids used in the present work are listed in Table 1. The *Escherichia coli* (*E*. *coli*) strains (DH10B and HST08—Stellar) used for cloning were cultivated in SOB and LB/LB-agar [20] at 37°C. *Xanthomonas citri* subsp. *citri* (*X*. *citri*) strain 306 were cultivated at 30°C in NB medium (“Nutrient Broth”: meat extract 3 g/L and peptone 5 g/L) or on NB-agar plates (NB medium containing agar 15 g/L) supplemented with L-arabinose (0.05% w/v) and starch 0.2% (w/v) when required. The antibiotics carbenicillin and kanamycin, when required, were used at the concentration of 50 μg/mL.

**Table 1:** List of strains and plasmids used in this work.

**Strains**	**Characteristics**	**References**
*X*. *citri* 306	*Xanthomonas citri* subsp. *citri* strain 306 (wild-type strain)	IBSBF 1594; Da Silva et al. [5]
*E*. *coli* DH10B	Cloning strain	Thermo Fisher Scientific, Waltham, MA, USA
*E*. *coli* HST08	Cloning strain	Takara Bio USA, Inc. Mountain View, CA, USA
**Plasmids**	**Characteristics**	**References**
pSG1194	*lacZ*; mCherry expression vector; Ap^R;^ Cp^R^	Feucht and Lewis [21]
pGCD21	*araC*-p*ara-gfpmut1*; *amy*106-912 Ap^R^; Km^R^; integrative vector in *X*. *citri*	Lorenzoni et al. [22] (GenBank KU678206.1)
pMAJIIc	Derivative of pGCD21, mCherry expression vector; Ap^R^; Neo^R^/Km^R^; *ara*C-p*ara;* integrative vector in *X*. *citri;*	This work (GenBank MT119765)

Ap^R^: ampicillin resistance, Cp^R^: chloramphenicol resistance, Km^R^: kanamycin resistance, Neo^R^: neomycin resistance

### Cloning procedures, vectors construction and mutant’s generation

**Fig 1:**
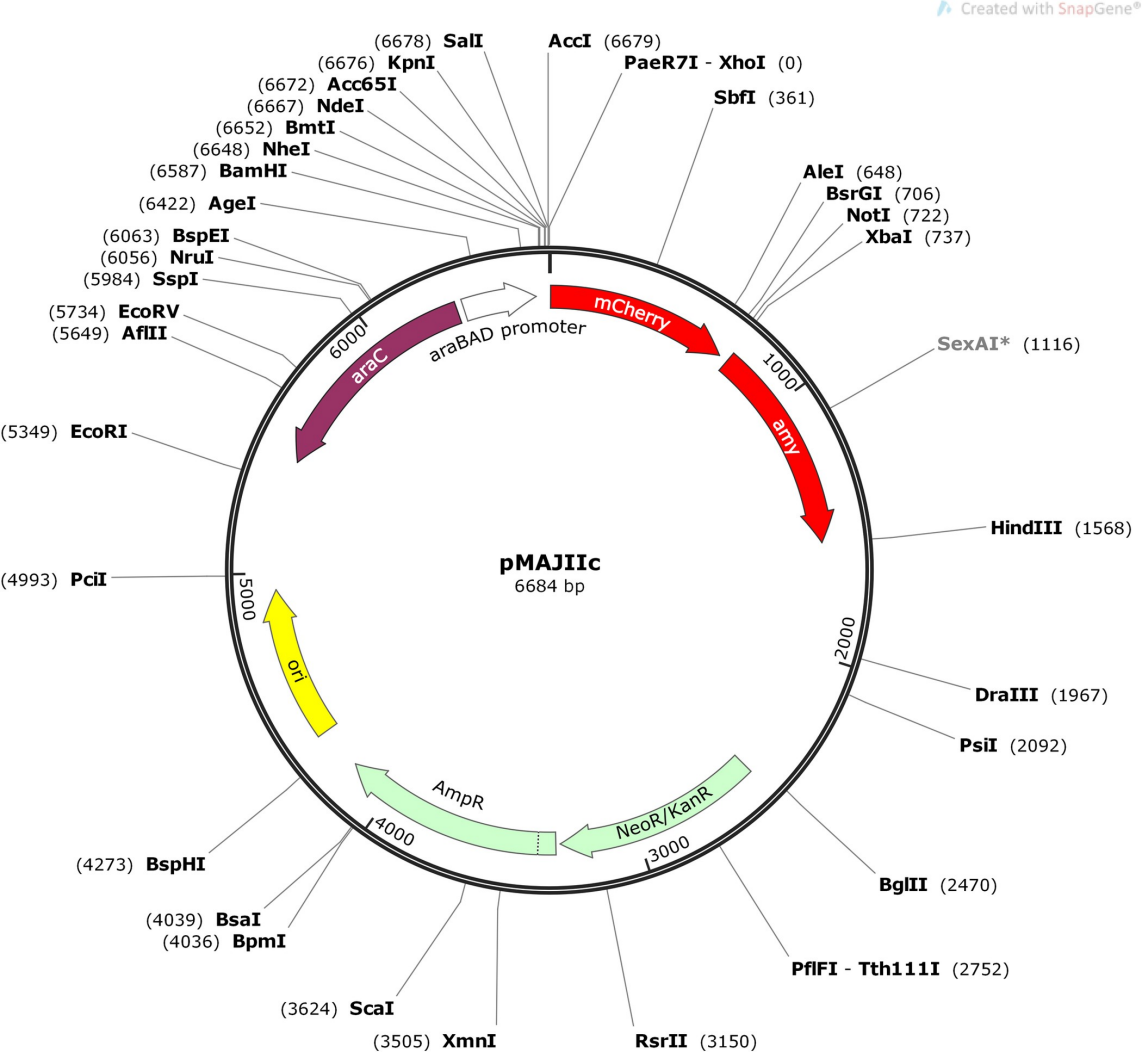
Map of the integrative vector pMAJIIc. *Ori*, replication origin; *ap*, *neo* and *kan*, ampicillin, neomycin and kanamycin resistance, respectively; *amy*, *amy* DNA fragment (106–912) of *X*. *citri*; *mCherry*, fluorescent protein; *araC*, the arabinose repressor; pBAD the arabinose promoter. The polylinker region for in-frame ligation of ORFs/genes to the 5’-end of mCherry, which allows the expression of protein fusions having C-terminal mCherry, is composed of unique sites for *Nhe*I, *Nde*I, *Kpn*I, *Sal*I and *Xho*I. *Not*I and *Xba*I sites, can be used for cloning intended for the expression of proteins carrying N-terminal mCherry fusions. Unique restriction sites are shown in bold letters. The nucleotide sequence of pMAJIIc plasmid was deposited in the GenBank database under the accession number MT119765.

The coding sequence for mCherry was PCR amplified from plasmid pSG1194 [21] using the primers mCherry_F and mCherry_R (Table 2) and Phusion high-fidelity DNA polymerase (New England Biolabs). The PCR-amplified fragment was cleaned up by phenol/chloroform extraction and ethanol-precipitation [20] and then digested with *Xho*I and *Not*I (New England Biolabs). The digested fragment was also cleaned up by phenol/chloroform extraction and ethanol-precipitation and then ligated (T4 DNA ligase) into pGCD21 plasmid [22] cleaved with *Xho*I and *Not*I (removes *gfpmut1* sequence), resulting in plasmid pMAJIc (GenBank MT119764). pGCD21 plasmid harbors a fragment of *X*. *citri amy* gene (bases 106–912), which allows the integration into *X*. *citri* chromosome by disrupting *amy* [22]. A complementary pair of oligonucleotides containing the restriction sites *Nde*I, *Kpn*I and *Sal*I were designed (primers pMAJ1_PL_F and pMAJ1_PL_R–Table 2) and subjected to hybridization, and the resulting double-stranded DNA (owning a 5’ *Nhe*I and a 5’ *Xho*I overhang cohesive ends) was ligated into pMAJIc upstream of the mCherry sequence (within the *Nhe*I/*Xho*I sites), giving rise to pMAJIIc plasmid (GenBank MT119765), which has a polylinker region containing the unique restriction sites *Nhe*I, *Nde*I, *Kpn*I, *Sal*I and *Xho*I (Fig 1). pMAJIc and pMAJIIc plasmid constructs were checked by DNA sequencing using primers pGCD21-F and pGCD21-R (Table 2).

**Table 2:** Primers designed for PCR amplification, DNA sequencing and qRT-PCR assay.

Name	Sequence
mCherry_F	5’-AA**CTCGAG**ATGGTGAGCAAGGGCGAGG-3’
mCherry_R	5’-TT**GCGGCCGC**CTGCTTCCTTGTACAGCTCGTCCATGC-3
pGCD21-F	5’-CACACTTTGCTATGCCATAGC-3’
pGCD21-R	5’-GATTACGACCAGTTCCTGGC-3’
pMAJ1_PL_F	5’-CTAGCAGGAGGAAAGAT**CATATG GGTACC GTCGAC**C-3’
pMAJ1_PL_R	5’-TCGAG**GTCGAC GGTACC CATATG**ATCTTTCCTCCTG-3’
VrpA_IF_F	5`-AGATCCATGGCA**CTCGAG**ATGCGATATCTACTGC-3’
VrpA_IF_R	5`-CTCACCAT**CTCGAG**CAGGTCTTGCTCGTATCTGACGTAC-3’
gyrB_IF_F	5`-AGATCCATGGCA**CTCGAG**ATGACCGACGAACA-3’
gyrB_IF-R	5`-GCTCACCAT**CTCGAG**GATATCCAGGTTGGACAC-3’
mCherry_RT_F	5`-CTGAGGTCAAGACCACCTACAAG-3’
mCherry_RT_R	5`-GTACTGTTCCACGATGGTGTAGTC-3’
gyrA_RT_F	5`-GTCAAGGAAAAGAAGCTCGAAG-3’
gyrA_RT_R	5`-GCTGATACAGGTTGTTGAGCAC-3’

F: forward primer, R: reverse primer. The sequences in bold represent the restriction sites. CTCGAG: XhoI; GCGGCCGC: NotI; CATATG: NdeI; GGTACC: KpnI; GTCGAC: SalI.

For the production of plasmids pMAJIIc-gyrB and pMAJIIc-vrpA, the *X*. *citri gyrB* gene sequence (XAC_RS00025) and the *X*. *citri vrpA* gene sequence (XAC_RS15480) were PCR amplified with the primers gyrB_IF_F and gyrB_IF_R and VrpA_IF_F and VrpA_IF (Table 2), respectively, using *X*. *citri* 306 genomic DNA as a template, and ligated into the *Xho*I site of pMAJIIc plasmid using the *In-Fusion HD Cloning Kit* (Takara Bio USA, Inc.) as recommended by the manufacturer. Both plasmid constructs were confirmed by DNA sequencing (primer pGCD21-F–Table 2) and, together with pMAJIIc alone, used to transform *X*. *citri* 306 strain to produce *X*. *citri* pMAJIIc (*X*. *citri amy*::pMAJIIc), *X*. *citri* pMAJIIc-*gyrB* (*X*. *citri amy*::pMAJIIc-*gyrB*) and *X*. *citri* pMAJIIc-*vrpA* (*X*. *citri amy*::pMAJIIc-*gyrB*).

### Integration stability assay

To confirm the integration and stability of the vector within the genome of *X*. *citri*, the mutant bacteria (*X*. *citri* pMAJIIc, *X*. *citri* pMAJIIc-*gyrB* and *X*. *citri* pMAJIIc-*vrpA*) were selected on NA plates supplemented with 0.2% of soluble starch followed by crystals iodine vapor exposure [23]. The stability was confirmed by keeping a continuous liquid bacterial culture without any antibiotic supplementation for seven days. For that, every day one milliliter from the overnight grown culture was transferred to a new 25 mL Erlenmeyer containing four milliliters of fresh NB medium; by the last day, the starch degradation assay was performed.

### Fluorescence microscopy

Bacteria were cultivated in 5.0 mL of NB medium for approximately 16 hours at 30°C and 200 rpm. The cultures were adjusted to the OD 600 nm of 0.1 with NB medium for a final volume of 5.0 mL and subsequently cultivated in the same conditions until the OD 600 nm of 0.3. At this point, arabinose was added to the medium to a final concentration of 0.05% and the culture was kept at 30°C and 200 rpm. After a minimum of two hours of induction, drops of 5 μL of cell culture were placed on microscope slides covered with a slide cover slip as described by Martins et al. [23]. Bacteria were immediately visualized using an Olympus BX61 microscope equipped with a monochromatic OrcaFlash2.8 camera (Hamamatsu, Japan) and TxRed filter. Data collection and analysis were performed with the software CellSens Version 11 (Olympus).

### Growth curves and pathogenicity assays

*X*. *citri* 306 and *X*. *citri* 306 harboring the plasmid pMAJIIc (*X*. *citri* pMAJIIc) were cultivated in NB medium for 16 h at 30°C and 200 rpm. For the *in vitro* growth curves, cultures were diluted in fresh NB medium to an OD 600 nm of 0.1 in a final volume of 1.5 mL (OD 600 nm of 0.3 correspond to 10^8^ CFU/mL). Cell cultures were distributed in the wells of a 24-wells microtiter plate and incubated in a microtiter plate reader (Synergy H1M1; BioTek) at 30°C with constant agitation (200 rpm) and OD 600 nm were measured every 30 min for 20 hours [24]. The *in planta* growth curves were performed according to procedures outlined by Zhang et al. [25], with modifications. The bacterial cultures were adjusted to 10^6^ CFU/mL with sterile 10 mM MgCl_2_ solution and infiltrated into the abaxial surface leaves of Valencia sweet orange (*Citrus sinensis*) using a needleless syringe. Samples for colony counting consisted of 1 cm^2^ leaf discs from the inoculation point at 0, 3, 6 and 9 DAI (days after inoculation), with the leaf discs being macerated in a 1.5 mL sterile Eppendorf tubes, in a final volume of 1mL of sterile 10 mM MgCl_2_ solution, with the help of sterile plastic pistils that fit perfectly into the tubes (micro tissue homogenizer), coupled to household drills. After a serial dilution by factors of 10, ranging from 10^−1^ to 10^−6^, it was plated on NA medium plates for colony count.

The pathogenicity tests were performed in triplicate using hosts with different susceptibility levels for *X*. *citri*: Valencia and Hamlin sweet oranges (*Citrus sinensis*), ‘Mexican’ lime (*Citrus aurantifolia*) and Rangpur lime (*Citrus limonia*). Bacterial cells (*X*. *citri* 306 and *X*. *citri* pMAJIIc) were adjusted to 10^8^ CFU/mL (OD 600 nm of 0.3) with sterile 0.9% NaCl solution and inoculate on the leaves abaxial surface using a needless syringe, as well as a negative control (NaCl 0.9%). Symptoms were observed for two weeks and were photo-documented at the 15^th^ day after inoculation.

### Western blotting and real-time Reverse Transcription PCR (qRT-PCR)

The expression of mCherry was evaluated by Western blot and qRT-PCR analysis using *X*. *citri* carrying the pMAJIIc plasmid. *X*. *citri* cells (*X*. *citri* pMAJIIc and *X*. *citri* 306) were cultivated in NB medium supplemented or not with arabinose 0.05%.

Total protein extracts were prepared by growing 3.0 mL of bacteria cultures for 16 h. The bacterial cells from 3.0 mL of each culture were pelleted by centrifugation (15000xg), 1 min in a microfuge) and 300 μL of Laemmli Buffer were added to each sample, heated at 95°C for 10 minutes and 30 μL were loaded onto two SDS-PAGE gels prepared following the instruction of TGX™ FastCast™ Acrylamide Solutions kit (BIO-RAD) and run in parallel.

For protein visualization, one gel was stained with Bio_safe^TM^ Coomassie G250 (BIO-RAD) for 30 minutes and destained for ~ 16 hours in distilled water; and for the Western blotting assay, the other gel was electroblotted onto a nitrocellulose membrane, washed with distilled water and incubated for ~ 2 hours with 5% skim milk in TBS (BIO-RAD). The polyclonal anti-mCherry primary antibody raised in rabbits (Bb pAb to mcherry 1:1000 abcam) was then added and incubated for ~ 16 hours in a cold room. After the incubation, the membrane was washed three times with TBS and the primary antibody was detected with anti-rabbit immunoglobulin raised in goat (goat pAb to Rb IgG HRP–abcam). Antibody detection and chemiluminescent reaction were performed according to the SuperSignal^TM^West Dura Extend Duration Substrate Kit (Thermo Fisher).

To obtain *X*. *citri* pMAJIIc and *X*. *citri* 306 total RNA, 1 mL of each bacteria culture (with and without arabinose as described above) were pelleted by centrifugation (15000xg), 1 min in a microfuge) and the RNA extraction followed the TRIzol^TM^ (Thermo Fisher) protocol. The first strand of complementary DNA was synthesized from 1 μg of total RNA using the qScript® cDNA SuperMix kit (Qiagen).

Primer Express 3.0 software (Applied Biosystems http://bioinfo.ut.ee/prime r3-0.4.0/) was used to design the primers for the mCherry gene (mCherry_RT_F and mCherry_RT_R) and the endogenous *gyr*A gene (gyrA_RT_F and gyrA_RT_R) used as control (Table 2).

The qRT-PCR was performed following the instructions for the SYBR Premix (Takara) using the primers in a final concentration of 1 μM and was run on a QuantStudio™ 3D Digital PCR System (Thermo Fisher Scientific, Waltham, MA, USA). The fold change of target genes expression was calculated using the Pfaffl method [26]. This experiment was repeated in triplicate with similar results.

## Results

### Stable integration of pMAJIIc vector into *X*. *citri* chromosome

**Fig 2:**
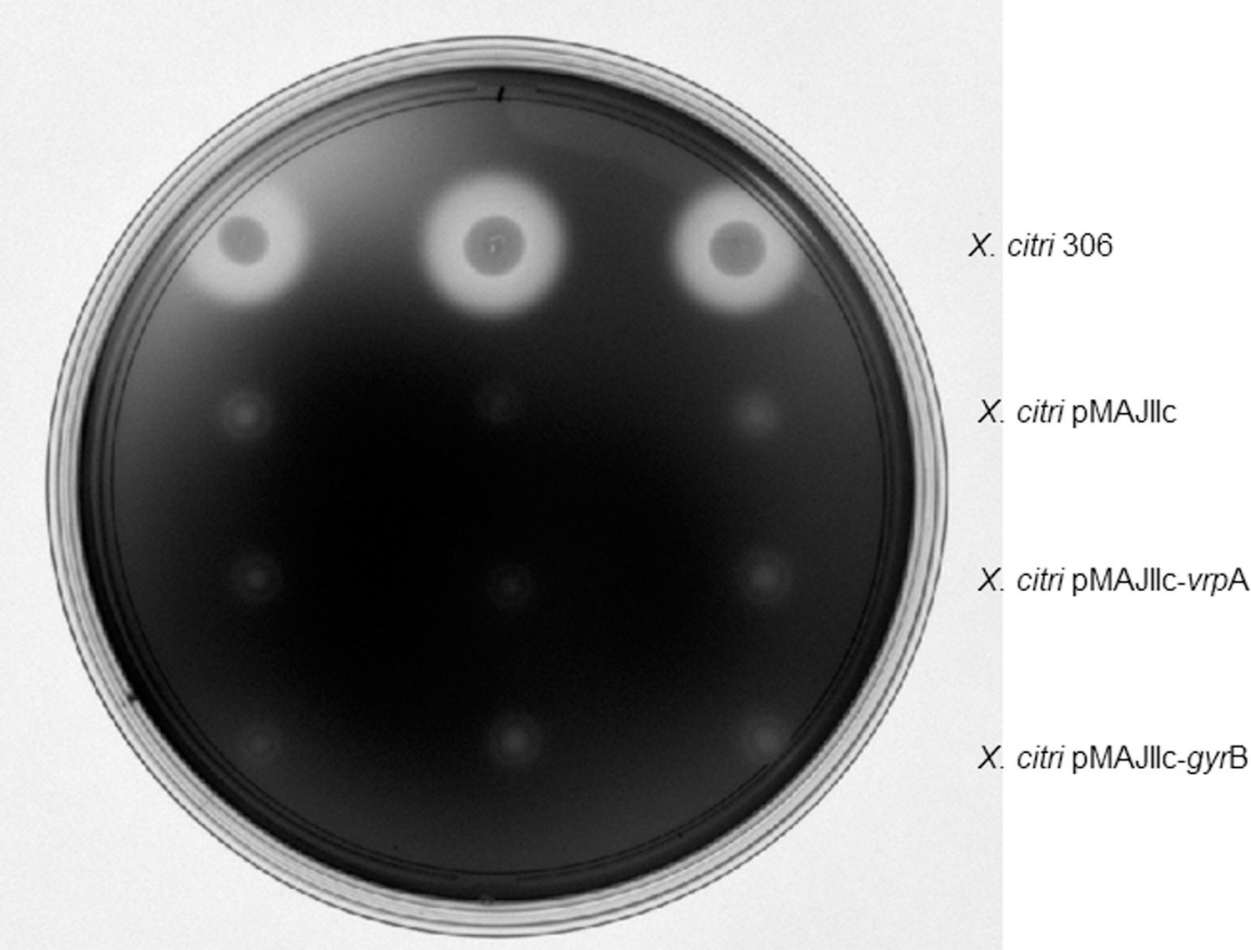
Starch degradation analyses. Halos corresponding to starch degradation were visualized by exposing the medium to iodine vapor. X. citri 306 produces a clear transparent halo (top), while mutants in which plasmids have integrated into the amy locus are unable to degrade the polymer (absence of halo).

An arabinose inducible integrative vector, pMAJIIc, was constructed to enable the visualization of gene expression and protein subcellular localization in *X*. *citri* (Fig 1). pMAJIIc is a suicide vector in *X*. *citri*, but it harbors a fragment of the *X*. *citri α-amylase* gene (*amy* gene; bases 106–914), which mediates its integration into the bacterial chromosome through a single crossover event. Since this integration disrupts the *amy* gene, the mutants produced are unable to degrade starch. Therefore, integration can be easily monitored by halo formation surrounding colonies on NA plates supplemented with starch (Fig 2). The vector also carries the arabinose promoter (pBAD) with its regulatory gene *araC*, a *mCherry* coding sequence and unique restriction sites allowing the in-frame ligation of mCherry to both ends of an ORF/gene intended for the expression of either N- or C-terminal mCherry protein fusions. To ensure the integration is stable, *X*. *citri* pMAJIIc was cultivated in liquid NB medium without antibiotics for a period of seven days. Bacteria were then screened on NA plates supplement with starch and the same phenotype was observed, with no halo formation surrounding the colonies.

### Subcellular localization of cytoplasmic and periplasmic proteins in *X*. *citri*

**Fig 3:**
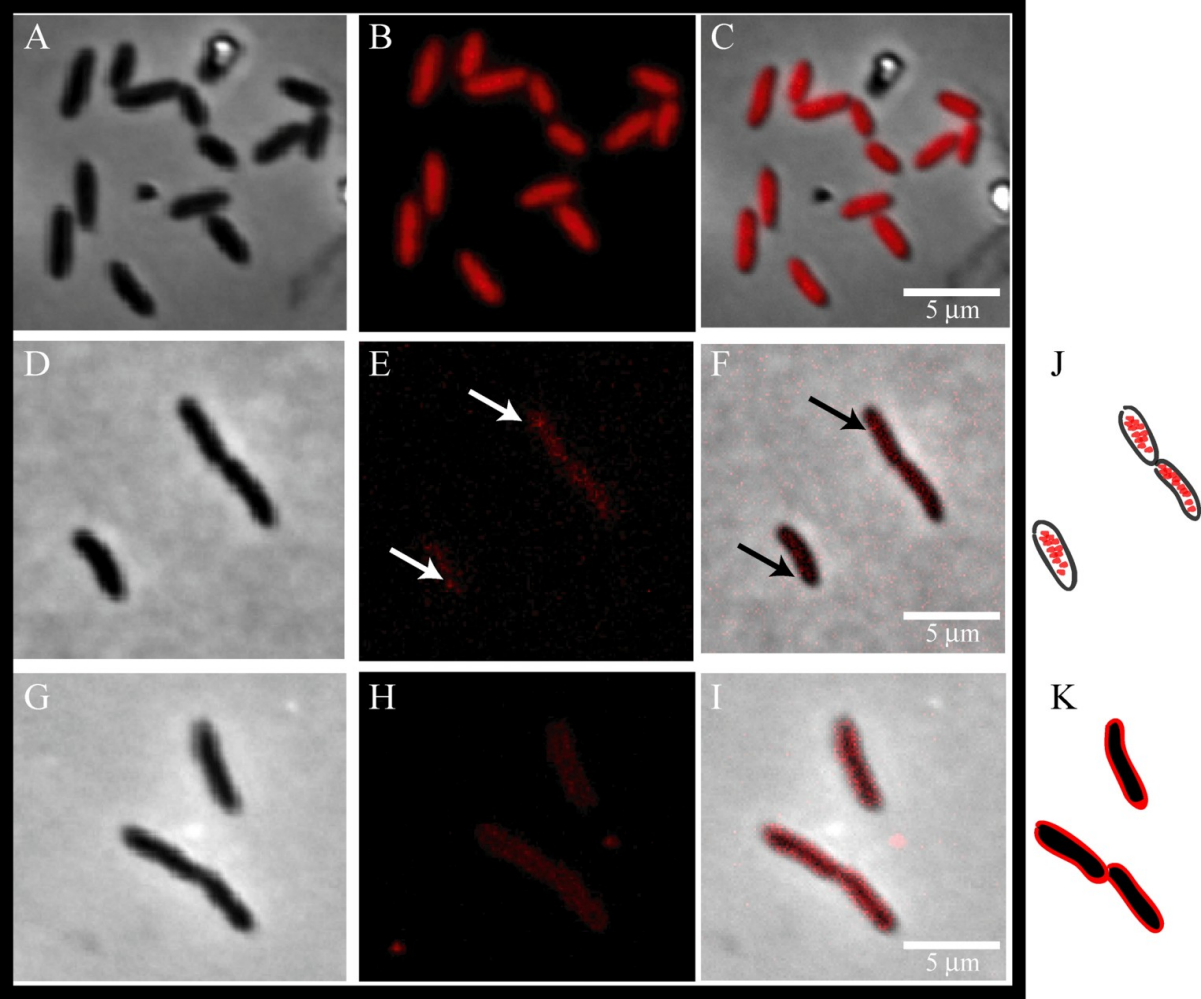
Subcellular localization of mCherry, GyrB-mCherry and VrpA-mCherry in *X*. ***citri*.** Mutant strains of *X*. *citri* expressing mCherry fusions were cultivated until the O.D.600nm of ~0.3, and subsequently induced with 0.05% arabinose for 2 hours prior to microscope observation. Panels show the phase contrast (left), TxRed channels (middle) and the overlay, respectively for each mutant: A-C: *X*. *citri* pMAJIIc (empty vector), D-F: *X*. *citri* pMAJIIc–*gyr*B and G-I: *X*. *citri* pMAJIIc-*vrp*A. The site of GyrB-mCherry accumulation is marked with a black arrow. J and K are representative cartoons for X. citri pMAJIIc–gyrB and X. citri pMAJIIc-vrpA, respectively. Magnification 100X; scale bar 5 mm.

To validate pMAJIIc as a tool for gene expression and the production of mCherry protein fusions in *X*. *citri*, we constructed *X*. *citri* mutants expressing C-terminal mCherry fusions to DNA gyrase B (GyrB-mCherry) and the virulence-related periplasmic protein A (VrpA-mCherry). The GyrB protein from *Bacillus subtilis* was shown to be bound to cytoplasmic DNA [27], while VrpA from *X*. *citri*, which carries a signal peptide, was reported to be localized in the periplasm [8]. First, *X*. *citri* cells expressing mCherry (*X*. *citri* pMAJIIc), the GyrB-mCherry (*X*. *citri* pMAJIIc-*gyr*B), and the VrpA-mCherry (*X*. *citri* pMAJIIc-*vrp*A) fusions all showed different fluorescence emission patterns after arabinose induction (Fig 3). *X*. *citri* pMAJIIc, which expresses mCherry alone, showed a strong and uniform fluorescence distribution spread all over the cytoplasm (Fig 3B and 3C). For the cells expressing GyrB-mCherry (Fig 3F), fluorescence was mostly observed in the same region where the bacterial chromosome is located (S1 Fig), e.g., centrally in the cytoplasm and not in the cell borders, as represented in the cartoon (Fig 3J). However, sometimes the GyrB-mCherry signal formed discrete foci near the cell pole (Fig 3F; arrows). Different from the fluorescence patterns showed by *X*. *citri* pMAJIIc cells, in *X*. *citri* pMAJIIc-*gyr*B the cells’ outer membrane in the red channel had a low signal (Fig 3E) and could only be identified in the phase and merge channel (Fig 3D and 3F). Finally, cells expressing VrpA-mCherry exhibited a fluorescence pattern that was consistent with the protein spread around the edges of the rod, while the cytoplasm remained non-fluorescent (Fig 3H and 3I), as represented in the cartoon (Fig 3K).

### Integration into *X*. *citri* chromosome does not affect bacteria pathogenicity and viability

**Fig 4:**
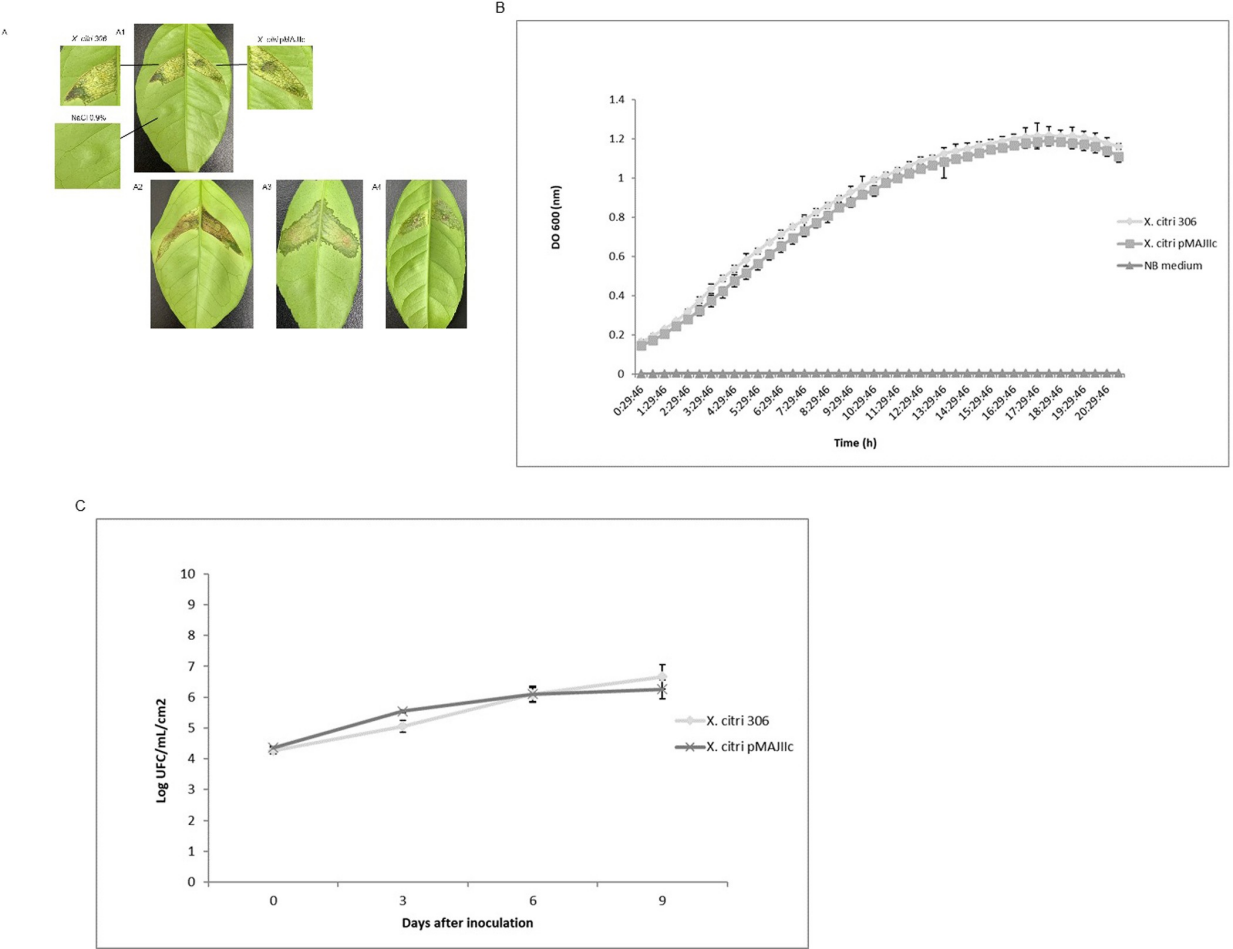
Pathogenicity assay and growth curves of *X*. *citri* 306 and *X*. *citri* pMAJIIc. A: Leaves of Valencia sweet orange (A1), Hamlin sweet orange (A2), Mexican lime (A3) and Rangpur lime (A4) were infiltrated with cell suspensions (10^8^ CFU/mL) of the indicated *X*. *citri* strains. All the inoculation followed the pattern displayed in A1. Pictures were taken at 15 days after inoculation (DAI). B: *in vitro* growth curve. *X*. *citri* pMAJIIc and *X*. *citri* 306 were cultivated in NB medium and OD 600 nm readings were taken every 30 min for 20 hours. C: *In planta* growth curve. Leaves of Valencia sweet orange were infiltrated with cell suspensions (10^6^ CFU/mL) of the indicated *X*. *citri* strains and bacterial populations were quantified at 0, 3, 6 and 9 DAI. All the experiments were done in triplicates.

The effect in the viability and pathogenicity of *X*. *citri* due to the integration of pMAJIIc vector into the *amy* locus of the bacterium was investigated. The development of citrus canker symptoms was evaluated in several citrus cultivars inoculated with *X*. *citri* pMAJIIc in comparison to *X*. *citri* 306 (positive control). The symptoms observed on the 15^th^ day after inoculation showed the borders and brownish lesions typical of citrus canker disease in both *X*. *citri* and *X*. *citri* pMAJIIc strains (Fig 4A). This result demonstrates that the integration of the plasmid pMAJIIc into the *amy* locus did not affect the ability of *X*. *citri* to cause citrus canker. A similar result was found by Lorenzoni et al. [22] with the plasmid pGCD21, which was used as backbone for pMAJIIc.

Besides, *in vitro* and *in planta* growth curves were performed to evaluate the growth capability of *X*. *citri* pMAJIIc strain in comparison with the *X*. *citri* 306 (Fig 4B and 4C). The *in vitro* growth curve showed that both strains grown equally along the time with no difference between *X*. *citri* pMAJIIc and *X*. *citri* 306 bacteria (Fig 4B). According to the curve, both strains follow the same lag and log-phases and by the 17^th^ hour started the declined phase. *In planta*, *X*. *citri* pMAJIIc and *X*. *citri* 306 strains showed similar development with no significant difference in colonization (Fig 4C). Different from the *in vitro* growth curve, the strains showed a shorter lag and log-phases, showing a stable grown on the 6^th^ day after inoculation.

Together, these results confirm that *X*. *citri* pMAJIIc is as competent as the wild type *X*. *citri* 306 strain to induce disease symptoms, with no alterations observed in growth and viability of the cells, both *in vitro* and *in planta*. Therefore, pMAJIIc constitutes a functional tool for the characterization of genes involved in virulence and pathogenicity.

### Effect of arabinose on the expression of mCherry

**Fig 5:**
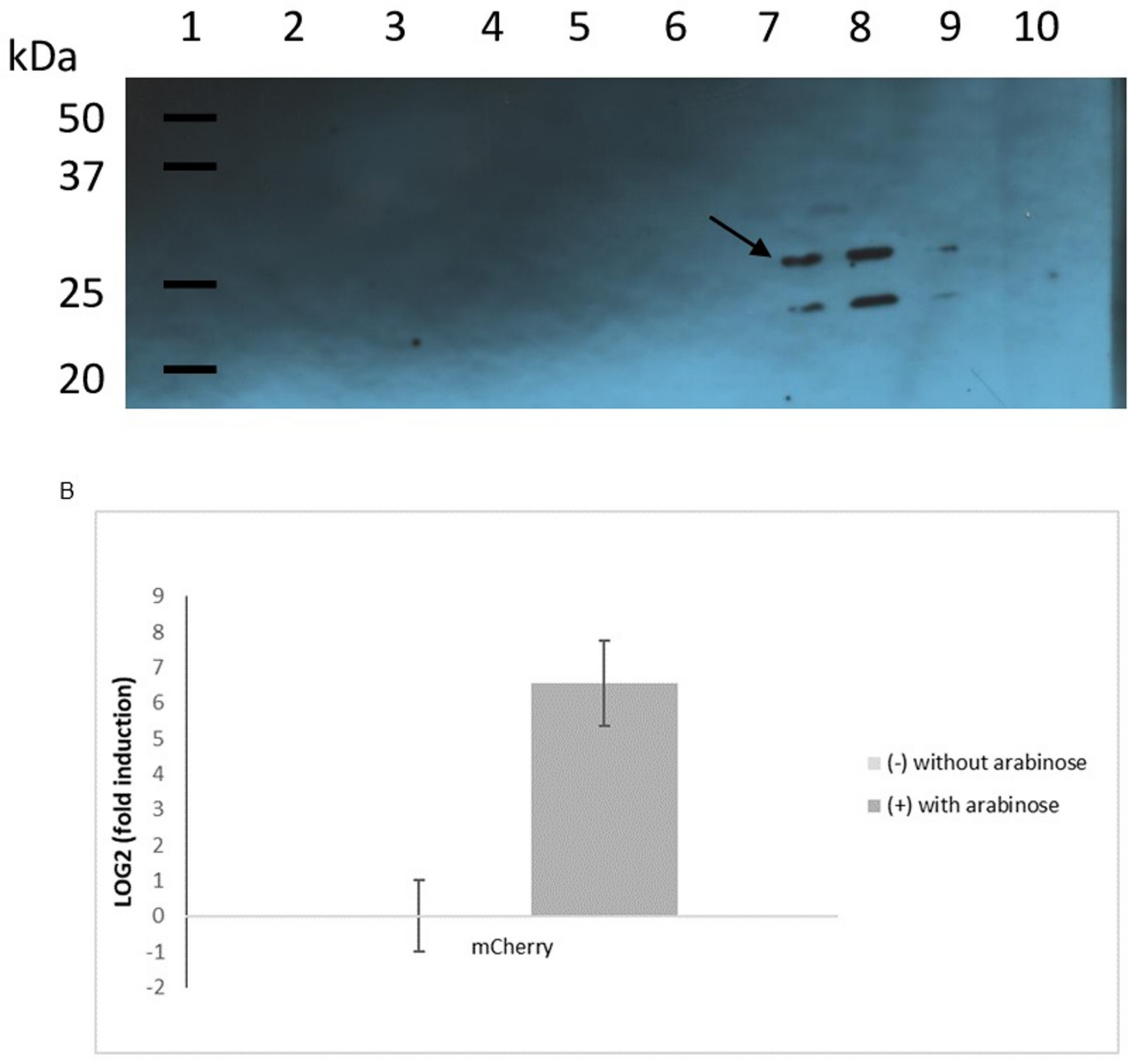
Effect of arabinose on the expression of mCherry. A: immunoblotting detection of mCherry expressed by *X*. *citri* pMAJIIc strain. For the Western blotting, six single colonies of *X*. *citri* pMAJIIc (harboring the mCherry expression cassette) were cultivated in NB medium and, for three of them, 0.05% arabinose was added to induce the mCherry production. *X*. *citri* 306 strain was used as control, and like the *X*. *citri* pMAJIIc strain, it was cultivated in NB medium with and without arabinose supplementation. Lane 1: molecular weight markers (Precision Plus Protein WesternC Standards–BioRad); lanes 2 and 3: *X*. *citri* 306 with and without arabinose supplementation, respectively; lanes 4, 5 and 6: *X*. *citri* pMAJIIc without arabinose supplementation; lanes 7, 8 and 9: *X*. *citri* pMAJIIc supplemented with 0,05% arabinose, lane 10: empty. A band corresponding in size to that expected for mCherry is marked by an arrow. B: mCherry expression level of *X*. *citri* pMAJII strain measured by qRT-PCR. No expression was observed in the untreated sample. The bars represent average ± SD of three experiments performed in triplicate with two replicates for each sample.

The expression of mCherry under the control of arabinose promoter was checked in *X*. *citri* pMAJIIc both by assessing the protein level by Western blot and the mRNA level by qRT-PCR in the presence and absence of arabinose (**Fig** 5). In the Western blot, a band of the expected size for mCherry (~29 kDa) was detected for the three *X*. *citri* pMAJIIc samples treated with arabinose (**Fig** 5A, lanes 7, 8 and 9). No band could be visualized for the non-induced *X*. *citri* pMAJIIc cultures (**Fig** 5A, lanes 4, 5 and 6) nor for *X*. *citri* 306 (**Fig** 5A, lanes 2 and 3). The band below mCherry might be a mCherry fragment produced by the breakdown of the protein due to its overexpression. The qRT-PCR assay (**Fig** 5B) showed that the mCherry mRNA was only produced when arabinose was added to the medium, with the expression level of the *mCherry* transcript being more than 50-fold higher in arabinose supplemented NB media compared to NB. Taken together, these results confirm that there is no leakage in *X*. *citri* harboring the mCherry cassette and that the expression of the mCherry is completely controlled by the *ara*BAD promoter.

## Discussion

Here we demonstrated the application of an integrative vector (pMAJIIc) for expression and subcellular localization of mCherry fusion-proteins in *Xanthomonas* sp. Fluorescent proteins have been used in this bacterium for different purposes [28–31], but this is the first report about protein localization in the periplasmic region of *Xanthomonas* using mCherry as a fluorescent marker. The protein localization system developed in this work used the integrative pGCD21 vector [22] as the backbone for the construction of pMAJIIc vector.

*X*. *citri* mutants carrying the pMAJIIc vector integrated into their chromosomes were as pathogenic as the wild type strain. Moreover, mutants were able to maintain the mCherry expressing plasmid throughout several generations. Integration into *X*. *citri* chromosome is mediated by a single homologous recombination/crossover event that could occur between the ORF to be characterized or the *α-amylase* fragment in the vector and their similar regions in the bacterial chromosome. For the empty vector and all genes characterized here, integrations into the *amy* locus were selected to avoid the possibility of gene/protein inequality and to enable the control of the target gene with the inducer arabinose. Besides, the disruption of the *amy* locus provides a readily distinguishable transformed colony screening selection method [21, 23, 32].

The expression of the *mCherry* gene, and fusions, in pMAJIIc is governed by the *ara* promoter extracted from the pBAD system, where the levels of expression of the protein fusion can be finely modulated by the concentration of arabinose in the medium. Although in the absence of arabinose transcription may occur at undetectable levels [24, 33], *X*. *citri* strains carrying the pMAJIIc plasmid did not show any leakage and the mCherry expression was only detected when arabinose was added to the medium. The arabinose system is well characterized in *E*. *coli* and the AraC protein is both a positive and negative regulator [34]; however, the efficient expression/repression depends on several factors like arabinose concentration, the ability to degrade arabinose, and the physiological state of the culture associated to the type of medium and carbon sources [35]. Guzman et al. [35] evaluated different strains of *E*. *coli* and different growing media and similar results were achieved. According to their results, the functionality of pBAD can be improved by using alternative compounds.

Fluorescent protein tags have been widely applied in protein localization and gene expression in rod-shaped bacteria studies and it has brought new insights into the cell biology of these microorganisms [36]. Some of these fluorescent proteins contain at least one cysteine residue, and to produce the fluorescence it must be correctly folded, otherwise there is no autocatalytic chromophore formation reaction [37]. Subcellular spaces, like periplasm of Gram-negative bacteria, are oxidizing environments that promote disulfide bond formation and it is highly unlikely that fluorescent proteins could achieve the necessary chromophore formation structure if cysteine residue incorporates into those bonds [37, 38]. To avoid this problem, a new monomeric protein family–mFruit–was developed. These proteins are more tolerant to N-terminal fusions, photostability, maturation and had its cysteine binding mutated to histidine, therefore the possibility of oxidation was eliminated [16].

Fluorescence microscopy has played an important role in determining the physiology and activity of cells, its structure and biological phenomena [39]. In this work, we were able not only to detect the expression of functional mCherry fusion proteins in *X*. *citri* cells grown in liquid culture but also to show their periplasmic localization. Two different proteins from *X*. *citri* were selected based on previous data about their cellular localization in *X*. *citri* itself or in other microorganisms’ homologs. The cytoplasmic protein GyrB is a type II topoisomerase that can supercoil and uncoil DNA helix during DNA replication, transcription and recombination [40]. In *X*. *citri* cell, GyrB-mCherry was visualized spread all over the cytoplasm with discrete foci accumulation close to the cellular pole. As showed by Ucci et al. [31], chromosome replication in *X*. *citri* starts in one of the cell poles and it is asymmetric, meaning that the protein was localized in the correct position, probably attached to the bacterium chromosome. Tadesse and Graumann [27] described the subcellular localization of *Bacillus subtilis* gyrase as not static but very dynamic. Gyrase was present on the nucleoids, showing accumulation in *B*. *subtilis* growing cells, although the accumulation assembled and disassembled in a few minutes [27]. Our result, indicating that GyrB of *X*. *citri* is a cytoplasmic protein and probably attached to the bacterium chromosome, which agrees with the findings of Tadesse and Graumann [27] for the subcellular localization of *Bacillus subtilis* GyrB protein. Besides, this is the first time that *X*. *citri* GyrB homolog is shown to be associated with the bacterial chromosome.

Fluorescence images from the construction pMAJIIc-*vrp*A in *X*. *citri* showed a strong emission surrounding the outer membrane of the bacteria cells, as expected for periplasmic proteins [41, 42]. This result is in accordance with previous findings of Zhou et al. [8], who shown that VrpA is a periplasmic protein in *X*. *citri* and it interacts with the periplasmic type-III secretion (T3S) system components HrcJ and HrcCo. The T3S system is part of the injectisome of Gram-negative bacteria, a hypodermic needle-like organelle responsible for delivering pathogenic effector proteins into host cells [43]. Wee and Hughes [44] showed the injectisome structure of *Samonella*, and according to the electron micrographs of the stained cells, this organelle is embedded in the cell wall spanning the periplasmic space between the inner and outer membranes.

The results presented here demonstrate that pMAJIIc vector, which allows the expression of in-frame N- or C- terminal mCherry-protein fusions under a controlled way, is able to show the fluorescence pattern and the subcellular localization for cytoplasmatic and periplasmatic proteins of *Xanthomonas citri* subsp. *citri* (*X*. *citri*). Although the vector was designed to integrate into *X*. *citri* genome, it could also be used for other Xanthomonas species, since the sequence of *amy* gene used for the genome integration (S2 Fig) shares more than 85% sequence identity with several other Xanthomonas species (S1 Table). Therefore, the vector described here can be a tool for bacterial subcellular protein localization, a step towards unlocking the function of hypothetical proteins.

## Supporting information

S1 Fig Nucleoid localization in *X*. *citri* 306. Bacteria were cultivated in 5.0 mL of NB medium until the OD 600 _nm_ of ~0.3 and cells were stained with DAPI (4′,6-diamidino-2-phenylindole) following the protocol described by Morão et al. [45]. Panels show the phase contrast (PhC), DAPI and the overlay. A: Image showing where the highlighted frame (B) was extracted to illustrate the standard positioning of the nucleoid in *X*. *citri*; B: phase contrast channel, C: DAPI channel, D: overlay B/C. Magnification 100X; scale bar 5 μm.
(TIF)

S2 Fig DNA sequence encoding the partial gene *amy* from *X*.*citri* 306 present in the plasmid pGDC21 (GenBank KU678206.1) and therefore also in plasmid pMAJIIc (GenBank MT119765). (TIF)

S1 Table Identity of the partial sequence of the *amy* gene of *Xanthomonas citri* subsp. *citri* 306 present in plasmid pMAJIIc in other *Xanthomonas* species. (PDF)

S1 Raw images (PDF)
