# Supplementary figures and images for: mCherry fusions enable the subcellular localization of periplasmic and cytoplasmic proteins in Xanthomonas sp
Source: PLoS One. 2020 Jul 30;15(7):e0236185. doi: 10.1371/journal.pone.0236185 (PMC7392301; doi:10.1371/journal.pone.0236185)

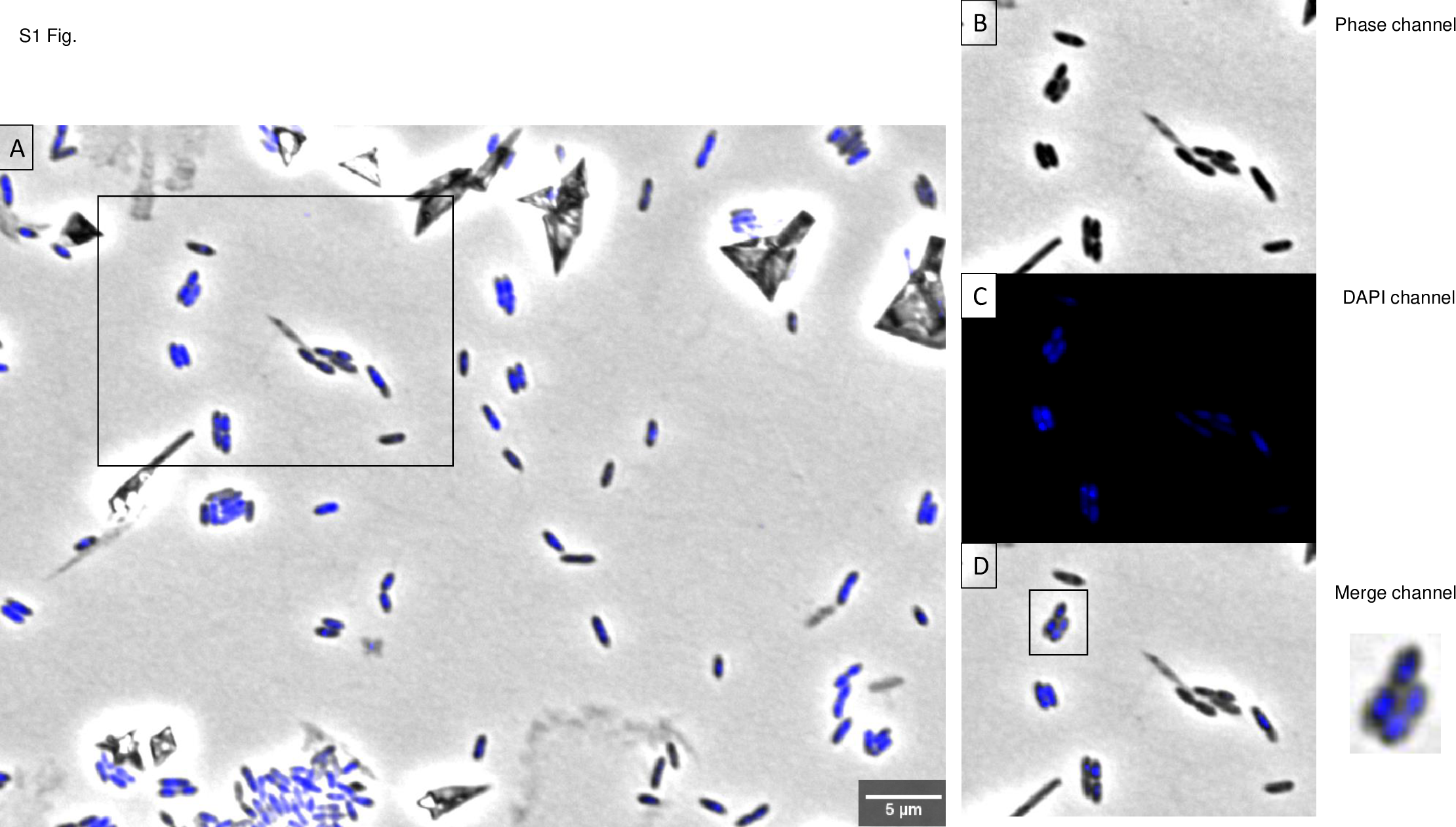

Supplement: S1 Fig — Bacteria were cultivated in 5.0 mL of NB medium until the OD 600 nm of ~0.3 and cells were stained with DAPI (4′,6-diamidino-2-phenylindole) following the protocol described by Morão et al. [45]. Panels show the phase contrast (PhC), DAPI and the overlay. A: Image showing where the highlighted frame (B) was extracted to illustrate the standard positioning of the nucleoid in X. citri; B: phase contrast channel, C: DAPI channel, D: overlay B/C. Magnification 100X; scale bar 5 μm. (TIF) [file pone.0236185.s001.tif]

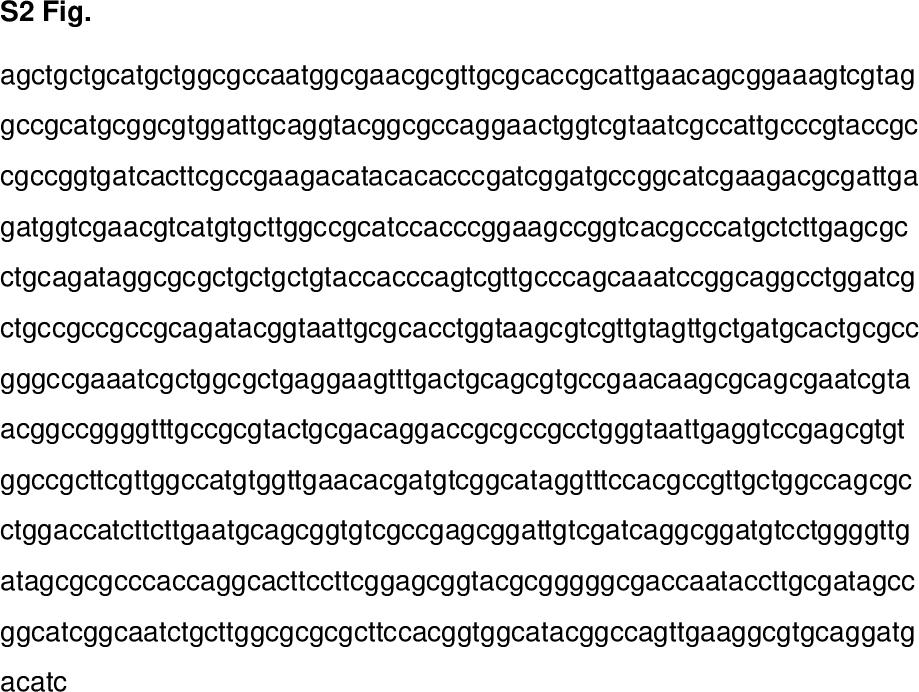

Supplement: S2 Fig — (TIF) [file pone.0236185.s002.tif]
