# Supplementary material for: mCherry fusions enable the subcellular localization of periplasmic and cytoplasmic proteins in Xanthomonas sp
Source: PLoS One. 2020 Jul 30;15(7):e0236185. doi: 10.1371/journal.pone.0236185 (PMC7392301; doi:10.1371/journal.pone.0236185)
Supplement: S1 Table — (PDF) [file pone.0236185.s003.pdf]

S1 Table.

**Species Description**	**% identity**	**Accession**
Xanthomonas citri subsp. citri A306, complete genome	100.00%	CP006857.1
Xanthomonas citri subsp. citri strain AW13	100.00%	CP009031.1
Xanthomonas citri pv. citri strain Xcc29-1 chromosome, complete genome	100.00%	CP023661.1
Xanthomonas citri pv. glycines strain K2 chromosome, complete genome	99.75%	CP041967.1
Xanthomonas citri pv. malvacearum strain HD-1 chromosome, complete genome	99.51%	CP046019.1
Xanthomonas citri pv. vignicola strain CFBP7113, complete genome	98.39%	CP022270.1
Xanthomonas citri pv. fuscans CFBP 6988 chromosome	98.27%	CP026331.1
Xanthomonas citri pv. phaseoli var. fuscans strain CFBP6991 chromosome, complete genome	98.27%	CP021015.1
Xanthomonas citri pv. anacardii CFBP 2913 chromosome	98.15%	CP024057.1
Xanthomonas fuscans subsp. aurantifolii strain FDC 1561, complete genome	98.15%	CP011250.1
Xanthomonas fuscans subsp. fuscans strain ISO118C5, complete genome	98.02%	CP012051.1
Xanthomonas perforans strain LH3 chromosome, complete genome	94.93%	CP018475.1
Xanthomonas axonopodis pv. commiphoreae strain LMG26789 chromosome, complete genome	94.81%	CP031059.1
Xanthomonas euvesicatoria strain LMG930, complete genome	94.56%	CP018467.1
Xanthomonas campestris pv. vesicatoria complete genome	94.56%	AM039952.1
Xanthomonas axonopodis pv. citrumelo F1, complete genome	94.31%	CP002914.1
Xanthomonas arboricola strain 17, complete genome	86.16%	CP011256.1

Xanthomonas arboricola pv. pruni strain 15-088 chromosome, complete genome	85.89%	CP044334.1
Xanthomonas arboricola pv. juglandis strain Xaj 417 genome	85.78%	CP012251.1
