# Supplementary material for: mCherry fusions enable the subcellular localization of periplasmic and cytoplasmic proteins in Xanthomonas sp
Source: PLoS One. 2020 Jul 30;15(7):e0236185. doi: 10.1371/journal.pone.0236185 (PMC7392301; doi:10.1371/journal.pone.0236185)
Supplement: S1 Raw images — (PDF) [file pone.0236185.s004.pdf]

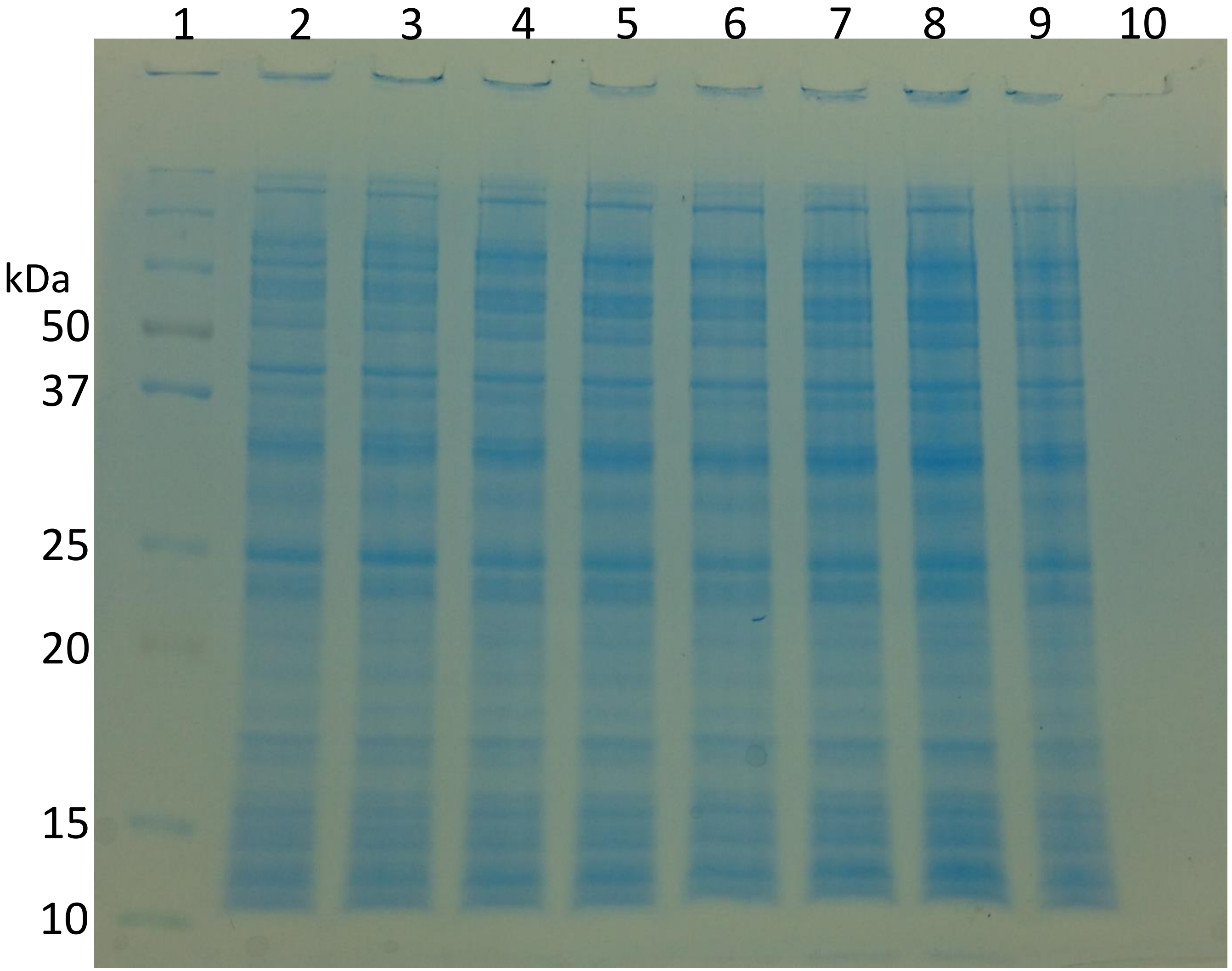

Samples in the wells :

Lane 1: Molecular Marker
Lane 2: *X. citri* 306 + arabinose
Lane 3: *X. citri* 306
Lane 4: *X. citri* pMAJIIc
Lane 5: *X. citri* pMAJIIc
Lane 6: *X. citri* pMAJIIc
Lane 7: *X. citri* pMAJIIc + arabinose
Lane 8: *X. citri* pMAJIIc + arabinose
Lane 9: *X. citri* pMAJIIc + arabinose
Lane 10: Empty

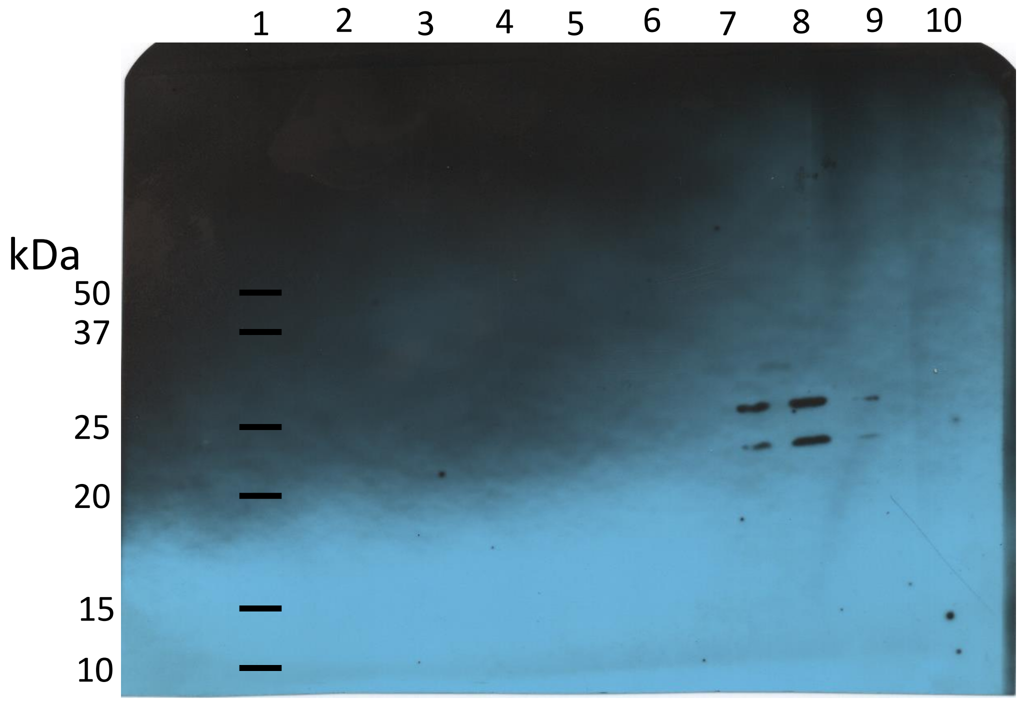

Samples in the wells:

Lane 1: Molecular Marker
Lane 2: *X. citri* 306 + arabinose
Lane 3: *X. citri* 306
Lane 4: *X. citri* pMAJIIc
Lane 5: *X. citri* pMAJIIc
Lane 6: *X. citri* pMAJIIc
Lane 7: *X. citri* pMAJIIc + arabinose
Lane 8: *X. citri* pMAJIIc + arabinose
Lane 9: *X. citri* pMAJIIc + arabinose
Lane 10: Empty

S1_raw_images: Comassie and Western Blot raw images were used to generate Figure 5A
